# Recombinant sterol esterase from *Ophiostoma piceae*: an improved biocatalyst expressed in *Pichia pastoris*

**DOI:** 10.1186/1475-2859-11-73

**Published:** 2012-06-07

**Authors:** Víctor Barba Cedillo, Francisco J Plou, María Jesús Martínez

**Affiliations:** 1 Centro de Investigaciones Biológicas (CIB), Spanish National Research Council (CSIC), Ramiro de Maeztu 9, Madrid 28040, Spain; 2 Instituto de Catálisis y Petroleoquímica, Spanish National Research Council (CSIC), Marie Curie 2, Madrid 28049, Spain

**Keywords:** *Ophiostoma piceae*, Sterol esterase, *Pichia pastoris*, Catalytic efficiency, N-terminal, Aggregation behaviour

## Abstract

**Background:**

The ascomycete *Ophiostoma piceae* produces a sterol esterase (OPE) with high affinity towards *p*-nitrophenol, glycerol and sterol esters. Its hydrolytic activity on natural mixtures of triglycerides and sterol esters has been proposed for pitch biocontrol in paper industry since these compounds produce important economic losses during paper pulp manufacture.

**Results:**

Recently, this enzyme has been heterologously expressed in the methylotrophic yeast *Pichia pastoris,* and the hydrolytic activity of the recombinant protein (OPE*) studied. After the initial screening of different clones expressing the enzyme, only one was selected for showing the highest production rate. Different culture conditions were tested to improve the expression of the recombinant enzyme. Complex media were better than minimal media for production, but in any case the levels of enzymatic activity were higher (7-fold in the best case) than those obtained from *O. piceae*. The purified enzyme had a molecular mass of 76 kDa, higher than that reported for the native enzyme under SDS-PAGE (60 kDa). Steady-state kinetic characterization of the recombinant protein showed improved catalytic efficiency for this enzyme as compared to the native one, for all the assayed substrates (*p*-nitrophenol, glycerol, and cholesterol esters). Different causes for this were studied, as the increased glycosylation degree of the recombinant enzyme, their secondary structures or the oxidation of methionine residues. However, none of these could explain the improvements found in the recombinant protein. N-terminal sequencing of OPE* showed that two populations of this enzyme were expressed, having either 6 or 8 amino acid residues more than the native one. This fact affected the aggregation behaviour of the recombinant protein, as was corroborated by analytical ultracentrifugation, thus improving the catalytic efficiency of this enzyme.

**Conclusion:**

*P. pastoris* resulted to be an optimum biofactory for the heterologous production of recombinant sterol esterase from *O. piceae,* yielding higher activity levels than those obtained with the saprophytic fungus. The enzyme showed improved kinetic parameters because of its modified N-terminus, which allowed changes in its aggregation behaviour, suggesting that its hydrophobicity has been modified.

## Background

Sterol esterases (EC 3.1.1.13) hydrolyze fatty acid esters of sterol and are widespread in nature, being the human cholesterol esterase one of the best studied among this group of enzymes [1-3]. However, those from microorganisms are the most used for biotechnological purposes since they can be produced in bulk at low cost. Some examples of these are the esterases from the bacterium *Pseudomonas aeruginosa*[4] and the actinomycete *Streptomyces*[5,6], the enzymes LIP2 and LIP3 from the yeast *Candida rugosa*[7-14], or those from the filamentous fungi *Melanocarpus albomyces*[15] and *Trichoderma* sp. AS59 [16].

Most of them share a common structural backbone belonging to the structural superfamily of α/β-hydrolases, like esterases and lipases, where residues responsible for its catalytic activity are highly conserved and form the so-called catalytic triad Ser-Asp/Glu-His [17], being the serine residue the nucleophile responsible for the beginning of catalysis. For this reason, all these enzymes are also known as serine hydrolases.

They display a wide range of molecular mass, usually from 20 to 80 kDa, although enzymes with lower masses have been described, for example the 6.5 kDa cholesterol esterase from *Acinetobacter*[18]. Typically, these proteins show tertiary structure but dimeric, tetrameric, and even hexameric forms or more can be found because of aggregation phenomena giving these pseudo-quaternary structures [6,19-21].

Their high versatility convert lipase-type enzymes in the most important group of biocatalysts in biotechnology, being used in different industries like food, detergent, pharmaceutical, leather, textile, cosmetic, and paper [22,23]. In fact, these enzymes accounted for about 5% of the world enzyme market in the year 2000 [24].

Recombinant DNA technology, together with protein engineering techniques, facilitates obtaining high quantities of an interesting enzyme at low cost, which allows the production of tailor-made biocatalysts [25]. Heterologous expression is a good approach for the production of these enzymes. Different prokaryotic and eukaryotic host systems can be used with this aim, although the advantage of the latest is its ability to carry out post-translational modifications, which can be very important to achieve the expression of a functional recombinant protein. This is the case of the methylothrophic yeasts, that provide a great potential as factories using methanol as the only carbon source [26]. The genera *Hansenula*, *Pichia*, *Candida,* and *Torulopsis* have been described as methylothrophic yeasts, but *Pichia*, and specially *Pichia pastoris,* is probably the most exploited yeast for recombinant protein production [27,28]. It is a consequence of the knowledge on the genetic characterization of this yeast gained during the last four decades, being a highly successful system for the production of a variety of heterologous proteins for the last 25 years [29]. This fact, and the advantage of having its genome sequenced [30], will contribute to facilitate recombinant expression and, inclusively, to improve it through systems biology strategies. Among the benefits of using *P. pastoris* as host system for heterologous expression are remarkable: i) its easy genetic manipulation and the stability of its transformants, thanks to the integration of the gene of interest by homologous recombination in its haploid genome, ii) its simplicity to be cultivated and to grow in minimal, and so inexpensive, media at high cell densities with low levels of endogenous protein secretion, together with the ability to efficiently secrete heterologous proteins, iii) fermentation processes are scalable to industrial levels of production, iv) different strong promoters are available to overexpress the gene of interest producing high quantities of protein, v) multiple copies of the gene of interest can be inserted into its genome, and vi) it also performs many of the higher eukaryotic post-translational modifications as protein folding, proteolytic processing, disulphide bond formation and glycosylation [31].

The sterol esterase from the saprophytic fungus *Ophiostoma piceae* (OPE) was previously characterized [32], and its possible application for pitch biocontrol during paper pulp manufacturing studied and patented because of its capability to hydrolyze triglycerides and sterol esters [33,34]. However, the use of any enzyme for biotechnological purposes requires making use of high quantities of protein. In a preliminary study [35], this enzyme was successfully expressed in *P. pastoris* (OPE*) and in this work we tackled the optimization of its production, studied its kinetic constants using *p*-nitrophenol, glycerol, and cholesterol esters, as well as analysed the cause of its improved catalytic properties.

## Material and methods

### Chemicals

*p-*nitrophenyl butyrate and palmitate, glycerol esters, cholesterol esters, and polyoxyethylene 10 tridecyl ether (Genapol X-100) were purchased from Sigma. Triton X-100 (reduced form) was acquired from Fluka. All chemicals were of the purest available grade.

### Fungal strains and plasmids

*O. piceae* (CECT 20416) was grown in modified Czapeck-Dox medium at 28°C and 160 rpm. *E. coli* DH5α (Stratagene®) was grown in Luria-Bertani medium at 37°C and 150 rpm, and used for cloning and plasmid amplification. *P. pastoris* GS115 (*his*4 auxotrophy) (Invitrogen™) was used as host strain for expressing the *O. piceae* mature esterase sequence under the transcriptional control of the *AOX*1 promoter (P_*AOX*1_). The vectors pGEM-T Easy (Promega) and the shuttle vector pPIC9 (Invitrogen™) were utilized for cloning and expressing the esterase gene, respectively.

### Plasmid and strain construction

The mature *O. piceae* esterase sequence, harbouring 5′ *EcoR*I and 3′ *Not*I sites previously incorporated by PCR, was cloned into the pGEM-T Easy vector, sequenced, and subcloned into the same sites of the pPIC9 vector. The construction was subjected to restriction analysis and sequenced to confirm the correct insertion and orientation of the mature esterase sequence. As a result, the OPE mature sequence was fused to the coding sequence of the α-mating factor secretion signal pre-propeptide for the extracellular release of the gene product. Transformation of *Pichia* spheroplasts with the *Sac*I linearized recombinant vector pPIC9OPE, allowed isolation of Mut^+^ phenotype transformants after homologous recombination of the vector within the 5′ *AOX*1 region in the yeast’s genome.

Oligonucleotides were synthesized by the Protein Chemistry facility at CIB. DNA sequencing was performed at SECUGEN.

### Culture conditions

*E. coli* DH5α was grown in Luria-Bertani medium (10 g/L tryptone, 10 g/L NaCl, 5 g/L yeast extract, and 15 g/L agar for solid medium) supplemented with 100 or 50 μg/mL ampicillin for liquid medium and agar plates, respectively. Bacteria were incubated at 37°C and 150 rpm overnight.

*P. pastoris* His^+^Mut^+^ transformants were screened for esterase activity in Petri dishes containing minimal methanol medium (MM) with tributyrin (13.4 g/L yeast nitrogen base without amino acids, 4 × 10^-4^ g/L biotin, 5 g/L methanol, 1% tributyrin, and 15 g/L agar). Plates were incubated during 48 h at 28°C and 100 μL of pure methanol were added daily to the lid of the inverted plates. Positive clones were initially checked in 1L Erlenmeyer flasks containing 100 or 200 mL of BMMY medium (described below).

The best *P. pastoris* transformant was grown in different culture media. i) buffered glycerol-complex/buffered methanol-complex media (BMGY/BMMY): 10 g/L yeast extract, 20 g/L peptone, 13.4 g/L yeast nitrogen base without amino acids, 4 × 10^-4^ g/L biotin, 100 mM potassium phosphate buffer pH 6.0, and 10 g/L glycerol for BMGY medium and 5 g/L methanol for BMMY medium, ii) buffered minimal glycerol/buffered minimal methanol media (BMG/BMM): same composition as BMGY/BMMY without yeast extract and peptone, iii) minimal glycerol/minimal methanol media (MGY/MM): 13.4 g/L yeast nitrogen base without amino acids, 4 × 10^-4^ g/L biotin and 10 g/L glycerol for MGY medium or 5 g/L methanol for MM medium, and iv) buffered sorbitol-methanol- complex medium (YEPS): 10 g/L yeast extract, 20 g/L peptone, 10 g/L sorbitol, 100 mM potassium phosphate buffer pH 6.0, and 5 g/L methanol.

A single colony from fresh yeast extract peptone dextrose (YPD) plates (10 g/L yeast extract, 20 g/L peptone, 20 g/L dextrose, and 20 g/L agar) was used to inoculate 25 mL of BMGY, BMG or MGY media in 250 mL flasks, which were incubated overnight at 28°C and 275 rpm until an optical density at 600 nm (O.D._600_) value between 2-6 was reached. Then, cells were harvested by centrifugation and resuspended in 100 mL or 200 mL of BMMY, BMM or MM media to an O.D._600_ of 1, in 1L Erlenmeyer flasks, following the Invitrogen’s protocol [36]. YEPS medium was directly inoculated from a fresh single colony.

Media used for induction and expression were maintained at 28°C and 250 rpm during at least 96 h. In these media methanol was added daily at a final concentration of 5 g/L for maintaining the induction and counteract evaporation.

### Enzyme assay, protein determination, and biomass measurement

Enzyme activity was routinely measured by monitoring *p*-nitrophenol release from 1.5 mM *p*-nitrophenyl butyrate (*p*NPB) in 20 mM Tris-HCl pH 7.0 buffer at room temperature in a Shimadzu UV-160A spectrophotometer [32]. One unit of activity (1U) is defined as the amount of enzyme releasing 1 μmol of *p*-nitrophenol (ε_410_ = 15,200 M^-1^cm^-1^) per minute under the defined conditions. 20 mM citrate-phosphate-borate buffer was used for stability studies and optimum pH determination.

Protein concentration was determined by the Bradford microassay (BioRad) using bovine serum albumin as standard (Sigma).

*P. pastoris* biomass was expressed as measures of turbidity of the samples at O.D._600_. This absorbance was correlated with the equivalent dry cell weight (DCW) in g/L multiplying the observed absorbance value by a factor of 0.2.

### Enzyme purification

The native enzyme was purified from 15-day-old *O. piceae* cultures in modified Czapeck-Dox medium supplemented with 0.5% olive oil [32]. The recombinant protein was purified from 4-day-old cultures in YEPS medium. Cultures were centrifuged 30 minutes at 13,000 rpm and 4°C, and filtered. Culture filtrates were concentrated by ultrafiltration using both a Millipore Pellicon™-2 Miniholder, coupled with a Masterflex® pump, and an Amicon® devices, equipped with 5 and 3 kDa cut-off membranes, respectively. 0.5 M (NH_4_)_2_SO_4_ was added to concentrated samples and these were applied to a HiTrap Octyl Sepharose FF Cartridge (GE Healthcare) previously equilibrated with 0.5 M of the salt in 25 mM Tris-HCl buffer pH 7.0, by using an Akta HPLC system (GE Healthcare). Proteins were eluted with a linear decreasing gradient (0.5-0 M) of (NH_4_)_2_SO_4_ in the same buffer during 60 minutes and the sterol esterase, which remained bound to the gel at the end of the gradient, was released after addition of 0.2% (v/v) reduced Triton X-100 in milli-Q water. Fractions containing esterase activity were pooled, diluted below critical micellar concentration of Triton X-100 in 25 mM sodium phosphate buffer pH 6.0, and concentrated again as mentioned above.

### Molecular mass determination

Sodium dodecyl sulphate polyacrylamide gel electrophoresis (SDS-PAGE) was performed in a Mini-protean III unit (BioRad) using running gels at 7.5%. N-linked-carbohydrate content (%) was estimated by comparing the molecular mass of the native and the N-deglycosylated protein. Deglycosylation was done treating the thermally denatured esterase with 10 mU of Endoglycosidase H (Roche) in 50 mM acetate buffer pH 5.0 with 0.06% (v/v) SDS and 0.1 mM β-mercaptoethanol at 37°C overnight. Protein bands were visualized either with Coomassie R-250 or silver staining (depending on protein concentration).

The purified recombinant enzyme (20 pmol/μL) was analyzed by MALDI-TOF mass spectrometry. The protein was crystallized in a saturated matrix solution (sinapinic acid in 0.1% (v/v) trifluoroacetic acid in a 1:3 acetonitrile:water solution). Desorption, ionization, and analysis of the sample was accomplished in an Autoflex III MALDI TOF/TOF instrument (Bruker Daltonics) equipped with smartbeam, and analyzed using the positive ion linear mode. *Protein Calibration* II (Bruker) ranging from 20 to 70 kDa was used as external standard.

### Kinetic studies

Catalytic parameters were obtained for different *p*-nitrophenol, glycerol, and cholesterol esters.

For these studies, the hydrolysis of *p*NPB and *p*-nitrophenyl palmitate (*p*NPP) were assayed in 3 mL reactions containing 100 mM sodium phosphate buffer pH 7.0, with 0.15 M NaCl and 1% (v/v) Genapol X-100 using an Uvikon spectrophotometer with magnetic stirring and temperature control at 25°C. One unit of activity (1U) is defined as the amount of enzyme releasing 1 μmol of *p*-nitrophenol (ε_410_ = 15,200 M^-1^cm^-1^) per minute under the defined conditions.

The hydrolysis of glycerol and cholesterol esters was assayed titrimetrically in a pH-stat model DL50 (Mettler Toledo) using 0.1 N NaOH as titrant at 25°C and 30% stirring rate. The reactions were carried out in 1 mM Tris-HCl buffer pH 7.0, with 0.15 M NaCl and 5% (v/v) Genapol X-100 in a final volume of 20 mL containing the substrate, which was previously emulsified in the detergent. One unit of activity (1U) is defined as the amount of enzyme releasing 1 μmol of free fatty acid per minute.

Experimental data were fitted to hyperbolic Michaelis-Menten curves and statistically analyzed with Sigma Plot 11.0 software.

### Circular dichroism spectroscopy

200 μL samples of both the native and recombinant proteins at 0.1 mg/mL in 25 mM sodium phosphate buffer pH 6.0 were monitored using circular dichroism spectroscopy (CD) on a J-720 spectropolarimeter (Jasco), in 1 mm light path quartz cuvettes (HELLMA). Spectra were acquired in the amide band (195-260 nm) at room temperature at 20 nm/min, with 0.5 nm bandwidth and a 4 s time constant. The spectra were measured in quadruplicate, averaged, and baseline corrected by subtraction of a buffer blank. Far-UV CD spectra were analyzed on DichroWeb website, using the K2d algorithm [37].

### N-terminal sequencing and amino acid composition

N-terminal sequence of the recombinant protein was obtained by automated Edman degradation of 10 μg of purified sample using a Procise 494 instrument (*Applied Biosystems*).

Amino acid composition and potential amino acid modifications were determined, in duplicate, with a Biochrom 30 analyzer (Biochrom, UK) after hydrolyzing 10 μg of native and recombinant sterol esterases with 6 N HCl at 110°C during 24 hours in vacuum. Amino acids were separated by cationic exchange chromatography and derivatized with ninhydrin postcolumn. Norleucine was used as the internal standard and methionine sulfone (Sigma) as standard of protein oxidation.

### Analytical ultracentrifugation

Analytical ultracentrifugation was used to compare the aggregation behaviour of both the native and recombinant proteins. Purified proteins (200 μg/mL) in 25 mM sodium phosphate buffer, pH 7.0, with and without 1% (v/v) Genapol X-100, were used for these experiments. Measurements were performed in a XL-A analytical ultracentrifuge (Beckman-Coulter Inc.) equipped with UV-VIS detection optics, using an An50Ti rotor. Protein concentration was estimated by absorbance. Results were analyzed with SEDFIT (version 11.8) and HeteroAnalysis (version 1.1.33) softwares for sedimentation velocity and equilibrium experiments, respectively.

## Results

### Screening of esterase producing clones

Twenty randomly picked His^+^ transformants were checked for methanol utilization by plating onto MM and MD plates as previously reported [35]. All transformants presented a Mut^+^ phenotype and exhibited hydrolytic halos on MM-tributyrin plates because of their ability to hydrolyze the triglyceride. Five transformants were selected and cultured in liquid media in Erlenmeyer flasks, in order to check their production levels.

### Production in Erlenmeyer flasks

The five His^+^Mut^+^ selected transformants were grown in 1L Erlenmeyer flasks containing 200 or 100 mL of BMMY medium. Differences regarding culture volume were found, showing better production in flasks with 100 mL of medium, between 3- and 7-fold increase in productivity depending on the colony, respect to the levels reached in flasks with 200 mL.

**Figure 1:**
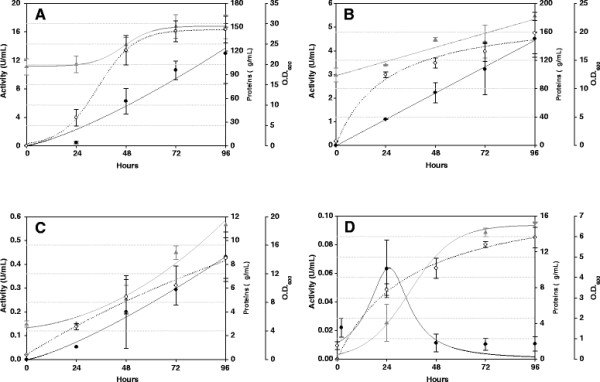
**Production of recombinant sterol esterase from *O. piceae *using different media. **Heterologous production in YEPS buffered complex medium with sorbitol as co-substrate (**A**), BMMY buffered complex medium (**B**), BMM buffered minimal medium (**C**.), and MM minimal medium (**D**). U/mL *vs p*NPB (black circle); proteins μg/mL (gray triangle); O.D. 600nm (white circle). Data points are the average of at least two different experiments. Error bars represent standard deviation. Data points were fitted to curves using Sigma plot 11.0 software.

The best transformant was selected to optimize esterase production using different culture media (Figure 1). Yields in buffered media (YEPS, BMMY, and BMM) were higher than in the unbuffered MM medium, where the activity was very low probably due to the drastic decrease in pH value (from pH 6.0 to pH 2.0 after 48 hours), which could affect enzyme activity. Complex media (YEPS and BMMY), with peptone and yeast extract, gave better yields than minimal media (BMM and MM). The addition of sorbitol as a second carbon source in YEPS medium was beneficial for the production. The sterol esterase activity levels in YEPS, BMMY, and BMM media, after 96 h of induction, turned out to be 7-, 2.5- and 0.2-fold higher than those got when *O. piceae* was cultured in modified Czapeck-Dox medium (1.8 U/mL) for 15 days [32]. The efficiency of *P. pastoris* cells on sterol esterase production in YEPS medium (~2,266 U/g biomass), was 2-, 15-, and 127-fold higher than in BMMY, BMM, and MM media, respectively.

### Purification and biochemical characterization

**Figure 2:**
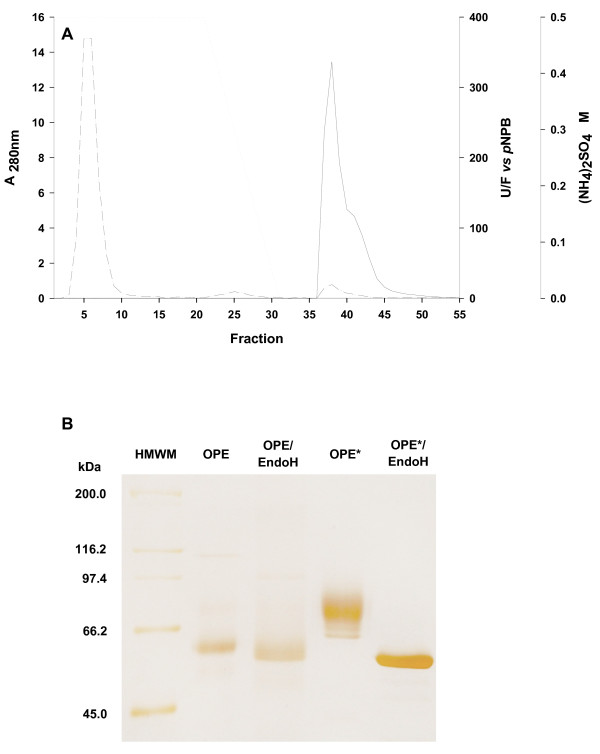
**Enzyme purification. **Hydrophobic interaction chromatography profile (**A**): units per fraction (___), A280nm (–·–·–·–) and gradient (········). SDS-PAGE for native and recombinant purified proteins before and after treatment with endoglicosidase H (**B**): High Molecular Weight Marker (HMWM), native *O. piceae *sterol esterase (OPE), and recombinant enzyme (OPE*). Protein bands are silver stained.

The recombinant protein was purified to homogeneity from 4-day old YEPS cultures by a single hydrophobic interaction chromatography step (Figure 2A) using an Octyl Sepharose cartridge (GE Healthcare), as previously reported [32]. Finally, the protein was dialyzed and concentrated, as mentioned above, and kept at -80°C remaining stable in these conditions at least for one year. 9 mg of pure enzyme were obtained from 1L of YEPS medium. The process yield was around 60% and protein was purified 9.2-fold (Table 1).

**Table 1:** Yield and purification grade throughout processing of recombinant enzyme

	**Activity (Units)**	**Protein (mg)**	**Yield (%)**	**Ae (U/mg)**	**Purification factor**
**Culture liquid**	9539	140	100	68	1.0
**Ultrafiltrate**	9839	89	103	111	1.6
**HIC-octyl sepharose and removal of Triton X-100**	5624	9	59	625	9.2

Ae: specific activity.

SDS-PAGE of the pure recombinant protein from YEPS cultures showed a band at *M*_*r*_ 76,000 Da, higher than that described for the native enzyme (60 kDa), and similar to that obtained from BMMY cultures [35]. The heterogeneity of the recombinant protein could be due to different patterns of post-translational modifications. This was confirmed after treatment with Endoglycosidase H (Figure 2B). The average molecular mass of the recombinant sterol esterase was found to be 74,921 Da by mass spectrometry and this value was used for the determination of its kinetic constants.

The effects of pH and temperature on enzyme activity and stability were investigated using 3 μg/mL aqueous solutions of the protein. The optimum pH of the purified recombinant protein was between 7.5 and 8.0, and its optimum temperature at 25°C, which differs from the value found for the native enzyme (around 60°C). Stability of the native and the recombinant enzyme was similar between pH 3.0 and pH 9.0, retaining around 50-70% of their initial activity after 24 h of incubation. Nevertheless, at pH 10.0 the recombinant enzyme maintained around 50% of activity while the native one kept less than 20%. Regarding temperature stability, OPE and OPE* maintained around 85% of their initial activity at 4°C and 30°C, after 24 h of incubation at pH 6. However, the recombinant protein was less stable than the native one at 45°C (50% and 35%, respectively) and both retained less than 10% of their activity at 60°C. It is interesting to mention that the stability towards pH and temperature of both proteins depends on their concentration, improving with increasing protein concentrations.

### Kinetic characterization

Reactions were carried out in presence of Genapol X-100 as surfactant due to the low solubility of most of the substrates assayed in aqueous solutions. Protein kinetic parameters were designated as “apparent” (app) as reported for native enzyme [32]. Table 2 summarizes the catalytic properties of the native and the recombinant esterase. Except for *p*NPB, the recombinant protein showed similar or higher affinity (lower *K*_m_^app^ values) than the native one, and greater turnover frequency (*k*_cat_^app^) in all cases. Its efficiency (*k*_cat_^app^/*K*_m_^app^) increased with the length of the acyl moiety in the substrate, as well as with the presence of insaturations, as reported elsewhere [32]. The catalytic efficiency of the recombinant enzyme, compared with the native esterase, was about 8-10-fold higher for all the substrates assayed.

**Table 2:** **Apparent kinetic parameters of native and recombinant sterol esterases from *O. piceae *** **on *p*-nitrophenol, glycerol and cholesterol esters**

**Substrate**	**Acyl length and insaturation(s)**	**Catalytic**	**Native**	**Recombinant**
		**parameters**	**OPE**^**a**^	**OPE**
***p*-Nitrophenyl butyrate**	C4:0	*K*_m_^app^	0.27±0.03	2.33±0.19
		*k*_cat_^app^	44±2	2533±55
		*k*_cat_^app^/*K*_m_^app^	162±13	1089±70
***p*-Nitrophenyl palmitate**	C16:0	*K*_m_^app^	0.33±0.03	0.37±0.03
		*k*_cat_^app^	74±3	1049±28
		*k*_cat_^app^/*K*_m_^app^	224±12	2875±212
**Glyceryl tributyrate**	C4:0	*K*_m_^app^	9.90±0.80	5.10±0.30
		*k*_cat_^app^	179±4	1041±14
		*k*_cat_^app^/*K*_m_^app^	18±1	204±10
**Glyceryl trioleate**	C18:1	*K*_m_^app^	0.98±0.08	0.71±0.09
		*k*_cat_^app^	290±7	1362±41
		*k*_cat_^app^/*K*_m_^app^	296±18	1924±197
**Cholesteryl butyrate**	C4:0	*K*_m_^app^	3.00±0.50	1.60±0.20
		*k*_cat_^app^	47±2	212±7
		*k*_cat_^app^/*K*_m_^app^	15.6±1.8	133±13
**Cholesteryl oleate**	C18:1	*K*_m_^app^	1.00±0.10	0.69±0.10
		*k*_cat_^app^	138±4	631±21
		*k*_cat_^app^/*K*_m_^app^	138±9	918±108
**Cholesteryl linoleate**	C18:2	*K*_m_^app^	0.99±0.06	0.71±0.09
		*k*_cat_^app^	150±3	798±25
		*k*_cat_^app^/*K*_m_^app^	152±6	1132±123

Reactions were carried out in the presence of the non-ionic detergent Genapol X-100.

*K*_m_^app^ (mM), *k*_cat_^app^ (s^-1^) and *k*_cat_^app^/*K*_m_^app^ (s^-1^mM^-1^). Standard errors are based on the curve fitting using Sigma plot 11.0 software. The *k*_cat_^app^/*K*_m_^app^ standard errors were obtained by fitting the normalized Michaelis-Menten equation as V = (*k*_cat_^app^/*K*_m_^app^)[S]/(1 + [S]/*K*_m_^app^). Kinetics parameters were obtained using the average molecular weight of the non-deglycosylated recombinant protein obtained by mass spectrometry as was previously considered with native one.

^a^[32].

### Effect of deglycosylation on recombinant enzyme activity

**Figure 3:**
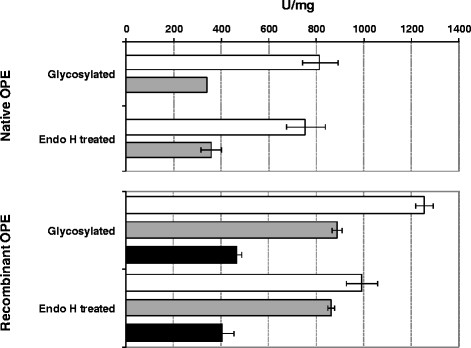
**Activity of native and recombinant enzymes before and after the deglycosylation treatment with Endo H. **Different substrates were assayed for the analysis: *p*NPB (white bars), triolein-TO (gray bars), and cholesteryl oleate-CO (black bars). Substrates were used at 5 mM concentration in the presence of Genapol X-100. Conditions are described in experimental section. Data points are the average of at least two different measures. Error bars represent standard deviation.

The higher degree of N-linked carbohydrate present in the recombinant protein (28% against 8% for the native protein) could contribute to improve its catalytic properties. Both esterases displayed the same *M*_r_ after deglycosylation with Endo H under non-denaturing conditions and did not show significant difference in their activities on *p*NPB, triolein, and cholesteryl oleate before and after deglycosylation (Figure 3).

### Far UV circular dichroism spectra

**Figure 4:**
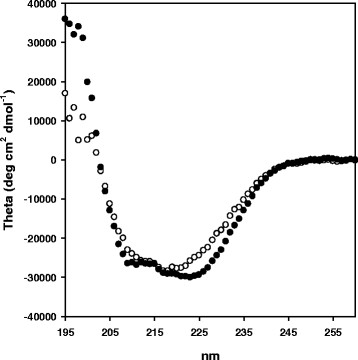
**Circular Dichroism spectroscopy. **Spectra of the native (white circle) and recombinant (black circle) proteins. Conditions are described in experimental section.

Secondary structure of the OPE and OPE* was analyzed by CD spectroscopy in order to find differences between them which could explain the kinetic results. However, their spectra were similar, with the two typical negative bands at 222 and 209 nm characteristic of α-helix (Figure 4). Analysis of the spectra by the K2d method from Dichroweb [37] resulted in an identical content of α-helix (0.46), β-sheet (0.23), and random coil (0.31) for both proteins.

### Methionine oxidation determination

**Figure 5:**
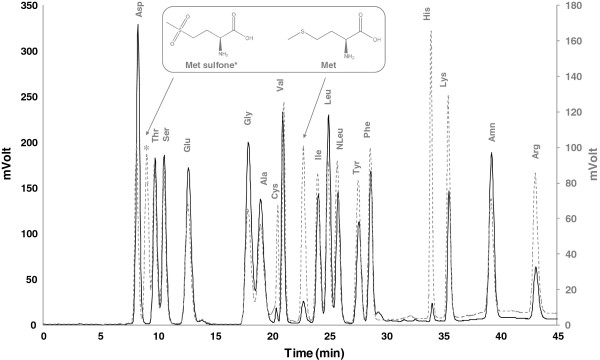
**Determination of oxidized methionine residues by amino acid analysis. **Chromatograms obtained from a hydrolyzed standard sample in which methionine sulfone was added (‐‐‐‐‐), and from a hydrolyzed sample of recombinant protein (____). Structures of methionine and methionine sulfone are showed in the inner box.

Oxidation in methionine residues has been previously reported in other proteins expressed in *P. pastoris*[38,39], and could be another factor to explain the improved catalytic properties of the recombinant enzyme. However, amino acid analysis of the recombinant protein revealed the lack of methionine sulfone, oxidized form of methionine (Figure 5).

### N-terminal sequencing

A modified N-terminal sequence was found in the recombinant enzyme (**EAEAYVEF**TTVNVNYPE) when compared to that from the native one (TTVNVNYPE). This fact can be explained because of the cloning process strategy, which added 4 amino acid residues (YVEF) belonging to the *Eco*RI (gaattc) and *Sna*BI (tacgta) recognition sites in the multiple cloning site (MCS) from pPIC9 vector. On the other hand, the incorrect processing of the pre-propeptide of the α-mating factor from *S. cerevisiae*, used as signal for secretion, by STE13 protease, adds two (EA) or four (EAEA) more residues to the sequence. Then, the N-terminal sequence of the OPE* contained 6 or 8 additional residues, and both forms were simultaneously found in the purified recombinant protein.

### Aggregation behaviour of native and recombinant enzymes

The aggregation state of both the native and recombinant proteins was studied by analytical ultracentrifugation techniques, comprising sedimentation velocity and equilibrium studies.

**Figure 6:**
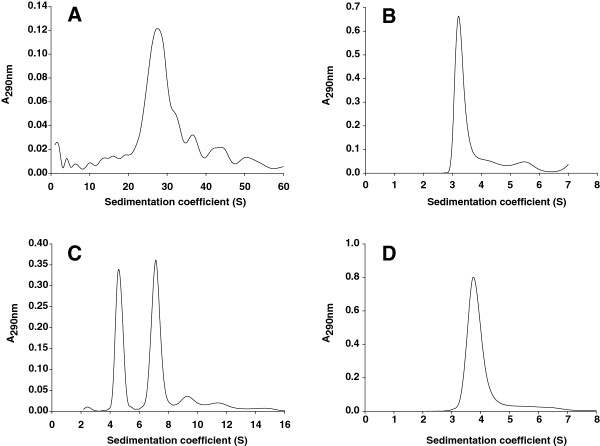
**Analytical ultracentrifugation. **Sedimentation velocities of the native (**A**) and recombinant (**C**) sterol esterases from *O. piceae* in aqueous solution and in the presence of Genapol X-100 (**B** and **D**, respectively). Conditions are described in experimental section.

Regarding the sedimentation velocity method, the native protein showed a high sedimentation coefficient (Figure 6A) which corresponded to a high molecular mass multi-aggregate in aqueous solution (25 mM sodium phosphate buffer pH 7.0), as previously reported [32]. However, under the same conditions, the recombinant protein presented sedimentation coefficients of 4.6S and 7.2S for the monomeric and dimeric forms, respectively (Figure 6C). When the study was performed in the presence of 1% (v/v) Genapol X-100, both proteins showed sedimentation coefficients of 3.3S and 3.8S, mainly compatible with the monomeric forms of the native and recombinant protein, respectively (Figure 6B and 6D). These coefficients differ to those measured in water solutions because the detergent affects the floatability of proteins. An additional experiment was carried out, in aqueous solution, with the deglycosylated OPE* obtained after treatment with Endo H in non-denaturing conditions. In this case, the protein was also found as monomeric and dimeric forms, with sedimentation coefficients of 4.2S and 6.7S, slightly lower than those obtained for the glycosylated protein because of the lack of N-glycan attached to asparagine residues.

Equilibrium experiments for glycosylated proteins in aqueous solutions corroborated an average molecular mass of 138 kDa (theoretical v-bar 0.73, being v-bar the partial specific volume) for OPE*, which is compatible with the dimer. No results were obtained for the native protein as a consequence of its tendency to form big aggregates, which prevents this kind of study. When a surfactant was employed, average molecular masses were 45 kDa (theoretical v-bar 0.73) and 56 kDa (theoretical v-bar 0.73) for OPE and OPE*, respectively (lower than those expected because of Genapol X-100, although compatible with the monomeric form). Concerning the deglycosylated recombinant protein, an average molecular mass of 109 kDa (theoretical v-bar 0.74) was obtained in aqueous solution. This can be explained from the coexistence of a mixture of molecular species, in contrast with the 47.5 kDa (theoretical v-bar 0.74) obtained for this sample in presence of the detergent, compatible with the monomeric form (data not shown).

## Discussion

Several advantages of using *P. pastoris* as biofactory for the production of the recombinant protein have already been mentioned. An additional reason to choose this system to express the *O. piceae* sterol esterase was that other fungal lipases have been successfully expressed in this yeast, such as the different isoenzymes (Lip1-Lip5) from *C. rugosa*[8,10,40-44] and *G. candidum*[45,46], as well as the sterol esterase from *M. albomyces*[15].

The screening to select the clones of *P. pastoris* with sterol esterase activity was carried out by using a simple plate activity assay [47] based on the hydrolysis of tributyrin in MM medium, which showed clear halos in the positive transformants. Initially, five positive clones were selected for the production of the sterol esterase in 1L Erlenmeyer flask containing different volumes of BMMY liquid medium. In any case, all clones secreted higher activity levels than those attained with *O. piceae*. However, the highest levels (up to 18 U/mL) were obtained when a lower volume of culture medium was used [35] because of the increase in the oxygen transfer rate. This is one of the different strategies previously proposed to facilitate oxygen transfer in Erlenmeyer flask cultures [28,31].

On the other hand, since the production of recombinant proteins in *P. pastoris* is closely connected to growth yields, the use of different carbon sources on biomass production, and so on the expression of the recombinant protein was studied. As methanol is a poor energetic substrate yielding in theory only 6 ATP molecules, both in assimilation and dissimilation pathways [48,49], the biomass production in media with methanol as the sole carbon source was lower than those obtained in media with sorbitol. In accordance with this, the biomass was lower for the selected *P. pastoris* Mut^+^ transformant growing in media with only methanol (MM, BMM, and BMMY) than in a medium with an alternative carbon source like sorbitol (YEPS), which do not repress *AOX1* promoter [50]. Consequently, the greatest activity levels were obtained in YEPS medium, being 3- and 32-fold higher than in BMMY and BMM media, respectively in terms of maximal activity. The performance of the production in YEPS medium, according to generated biomass, was higher than in BMMY and BMM (2- and 15- fold, respectively) and much higher than with MM (127-fold). The use of either complex or unbuffered media could contribute to decrease the effect of proteases during growing of *P. pastoris*. In the first case, yeast extract and peptone not only improve the growth of the yeast, but also can be substrates for proteases and could suppress their expression when nitrogen is limited [28,51]. In the second case, the growth of the yeast causes a fall in pH, favouring protease inactivation [36]. All these facts could explain the better activity levels found in BMMY medium as compared with BMM medium, and the very low activity detected in MM media. These results agree with previous reports describing higher activity levels of the recombinant cinnamoyl esterase from *Aspergillus niger* expressed in the yeast when buffered complex media were used [52]. Nevertheless, low activity levels of recombinant *M. albomyces* sterol esterase have been obtained in these culture conditions [15]. On the other hand, and also agreeing with our data, *G. candidum* lipase activity was not detected in cultures in MM medium [46], probably due to a pronounced decrease in pH with time (around 2), which would cause the denaturalization of the enzyme.

The recombinant enzyme was purified from YEPS medium in a single chromatographic step, with a purification factor higher than that obtained for the native enzyme [32]. LIP3 from *C. rugosa* was purified with a similar procedure, but yielding lower amounts of protein [8].

The purified enzyme can work in a wide pH range keeping more than 50% of its initial activity, as has also been reported for recombinant LIP3 [8], and it is thermostable at 4°C and 30°C for 24 hours in the assayed conditions. In any case, comparing the native and recombinant proteins, OPE* showed higher stability at very alkaline pHs and lower optimum temperature than OPE, which could be advantageous for its industrial application.

The existence of different post-translational modifications was considered in order to explain the observed changes, not only in the optimum temperature but also in the kinetic parameters of the recombinant enzyme. Regarding glycosylation, it has been described that long outer chains can potentially interfere with the folding and function of a foreign protein [29]. However, dichroism spectroscopy experiments (Figure 4) indicated that the recombinant protein was not misfolded, and an identical secondary structure was deduced for both sterol esterases. In addition, N-linked carbohydrates did not seem to be needed for maintaining the hydrolytic activity of these proteins, as deduced from deglycosylation experiments (Figure 3). Similarly, lipase B from *Candida antarctica*[53] and LIP4 from *C. rugosa*[43] maintained comparable kinetic properties after their expression in *E. coli*, although the glycosylated form of LIP4 produced in *P. pastoris* had higher thermal stability.

On the other hand, the partial oxidation of methionine residues during heterologous expression of proteins in *P. pastoris* has been reported [38,39]. Peroxisome environment in *P. pastoris* is highly oxidative and oxidation of sensitive residues could occur when hydrogen peroxide, produced during methanol metabolism, is released from peroxisomes to the culture medium after minimal cell lysis [39]. The sequence of the *O. piceae* sterol esterase contains 5 oxidizable methionine residues, one of them located in the surroundings of the substrate binding site [35]. However, amino acid analysis of the recombinant *O. piceae* sterol esterase suggested that this is not the reason for its improved catalytic properties, since no methionine sulfone residues were found (Figure 5).

Secretion is the preferred approach for heterologous protein production due to the ease of product recovery [54]. Furthermore, the secreted recombinant protein in *P. pastoris* constitutes the vast majority of total protein in the medium because the yeast secretes low levels of endogenous proteins [29]. However, the high level of expression from P_*AOX*1_ may overwhelm the post-translational machinery of the cell causing an unprocessed foreign protein [29]. The bad processing of the pre-propeptide of the α-mating factor can be explained by the formation of tertiary structures during the expression of a foreign protein that could protect cleavage sites from KEX2 and STE13 proteases [29]. In addition STE13, which cleaves EA repetitions, is a minor protein in the cell and it would not be able to process correctly an overexpressed protein [55]. Sequencing of the N-terminal region of the recombinant *O. piceae* esterase disclosed a wrong processing of the protein, since its N-terminus contained 6 or 8 additional residues from the secretion signal and the vector. This modification at the N-terminal end seems to influence some properties of the recombinant protein such as its aggregation state, as shown by analytical ultracentrifugation (Figure 6). While the native enzyme forms big aggregates in water solution [32], as reported for the *M. albomyces* sterol esterase [21], the recombinant enzyme remained as a mixture of monomeric and dimeric forms (even at 200 μg/mL). This behaviour could be the ultimate reason responsible for the improved catalytic properties of the recombinant enzyme. A wrong processing of the α-mating factor pre-propeptide has also been described in other proteins expressed in *P. pastoris,* such as the feruloyl esterase from *Talaromyces stipitatus*[56], the xylanase from *Thermomyces lanuginosus*[57], as well as the lipases from *C. antarctica*[53] and *Candida parasilopsis*[58], but the catalytic properties of these recombinant proteins were not affected. This wrong processing has been reported even in *S. cerevisiae*[59].

Esterases and lipases form pseudo-quaternary structures easily in aqueous solution. Multimeric forms have been described for the *M. albomyces* sterol esterase in the absence of detergent [21] although, at low concentrations, tetrameric forms have been reported for the native protein, and dimeric structures for the recombinant variant [15,60]. This tendency to form multimolecular aggregates has also been reported in the lipase from *C. parapsilosis*[58] and in the sterol esterases from *Streptomyces* species [6]. In addition, recombinant *C. rugosa* LIP2, expressed in *P. pastoris*, resulted in an aggregated, inactivated form of the protein, and only after diaultrafiltration lipolytic activity was recovered [10]. Monomolecular forms from *C. rugosa*, *Humicola lanuginosa* (synonym *T. lanuginosus*)*,* and *Mucor miehei* lipases were found only at low enzyme concentrations and in the presence of detergents. So it is difficult to find only the monomolecular form of lipase-type enzymes since it can only be achieved by mixing the enzyme solution with a detergent [61].

Lipases display different functional properties in their monomeric or aggregated forms [61]. In general, it seems that multimolecular forms exhibit lower specific activity and higher stability to pH and temperature than the monomeric proteins, although controversial data have been published for *C. rugosa* lipase [62]. For instance, enzymes from *M. albomyces* and *Streptomyces* sp. increased their activity in the presence of a detergent, where proteins probably tend to be in their monomolecular form [6,21]. However, in the case of *O. piceae* sterol esterase, neither activation nor inactivation of the enzyme (native or recombinant) has been reported in the presence of 0.2% Triton X-100 (used in the purification of these proteins), although above this concentration a decrease in their activity was observed (data not shown) as reported for the lipase BTL2 from *Bacillus thermocatenulatus*[63]. The use of Genapol X-100, which is indispensable in reactions involving long-chain triglycerides or fattyacid cholesterol esters in order to solubilise them, is detrimental for enzyme activity on *p*NPB since it acts as a competitive inhibitor for this short chain substrate [32]. In any case, as we report here, the use of detergents favour the monomeric form of the protein.

In accordance with previous works [61], when a concentrated aqueous solution of the recombinant enzyme was maintained during 16 h at 37°C no significant loss of activity was found. On the contrary, if this solution is diluted and the resulting solution incubated under the same conditions, an appreciable amount of activity was lost.

The high overall content in hydrophobic amino acid residues (38%) of the native enzyme could explain its tendency to form aggregates, as has been suggested for lipase BTL1 from *B. thermocatenulatus*[63]. However, the modification at the N-terminal end of the recombinant protein expressed in *P. pastoris,* by the addition of 6-8 extra amino acid residues from pPIC9 vector, used for protein expression, and the inefficient processing of the α-mating factor pre-propeptide, used for secretion, affected the aggregation state of the protein, as was confirmed by analytical ultracentrifugation experiments with deglycosylated recombinant enzyme.

Usually, a bad processing has no effect on recombinant protein activity, such as in *G. candidum*[46], *Yarrowia lipolytica*[64], and *C. parasilopsis* lipases [58]. An improvement of catalytic properties [65-69] and stability [8,42] of some recombinant and native enzymes has been previously reported, speculating on the basis of different glycosylation degree, N-terminal modification or aminoacid substitution (due to a preferential codon use in *P. pastoris* respect to natural host) [42]. However, to the best of our knowledge, the results presented in this paper constitute the first experimental report of an improvement of the solubility and kinetic constants of the enzyme, as a consequence of its N-terminal modification.

## Conclusions

The selection of the expression system is important in order to guarantee that a bioactive recombinant protein is produced with good yield. In this context, *P. pastoris* is a well-characterized system, which offers different possibilities for expression and is used in well-studied bioprocesses. So, it could be considered as an optimal biofactory for the production of sterol esterases, lipases, and esterases. This yeast resulted to be an excellent system for the heterologous production of recombinant sterol esterase yielding higher protein levels than those obtained with *O. piceae.* The recombinant protein showed different optimum temperature and improved catalytic properties probably due to its modified N-terminus, which must have caused changes in its hydrophobicity, altering its aggregation behaviour, and affecting positively its hydrolysis efficiency for all the substrates assayed.

## Competing interests

The authors declare that there are no competing interests.

## Authors’ contributions

VBC performed the DNA cloning and microbial transformations, the activity assays, characterization of the enzyme, data analysis, and drafted the manuscript. FJP designed the experiments for the kinetic characterization of the enzymes. MJM designed and coordinated the study and helped to draft the manuscript. All authors read and approved the submission of the manuscript.
